# Concentrated Distribution of Nicotine Patches to a Town With High Smoking Rates—Any Evidence of Large Impacts on Tobacco Cessation?

**DOI:** 10.1093/ntr/ntac244

**Published:** 2022-10-20

**Authors:** John A Cunningham, Scott T Leatherdale, Michael Chaiton, Rachel F Tyndale, Christina Schell, Alexandra Godinho

**Affiliations:** National Addiction Centre, Institute of Psychiatry, Psychology and Neuroscience, Kings College London, London, UK; Centre for Addiction and Mental Health, Toronto, Canada; Department of Psychiatry, University of Toronto, Toronto, Canada; School of Public Health and Health Systems, University of Waterloo, Waterloo, N2L 3G1, Canada; Centre for Addiction and Mental Health, Toronto, Canada; Dalla Lana School of Public Health, University of Toronto, Toronto, M5T 3M7, Canada; Centre for Addiction and Mental Health, Toronto, Canada; Department of Psychiatry, University of Toronto, Toronto, Canada; Department of Pharmacology and Toxicology, University of Toronto, Toronto, M5S 1A8, Canada; Centre for Addiction and Mental Health, Toronto, Canada; Centre for Addiction and Mental Health, Toronto, Canada

## Introduction

Rates of cigarette smoking can vary greatly between geographic regions.^1–3^ This pilot study examined whether there was benefit to the concentrated distribution of tobacco cessation resources—in this case the distribution of nicotine patches—to a region with higher-than-average smoking rates. The goal of this research was to contribute towards policy decisions regarding the efficient use of public resources to promote the largest number of people quitting smoking. If the study observed that offering free nicotine patches to all households in a community with higher-than-average smoking rates led to large reductions in smoking in the community, it would provide support for initiatives designed to target mass distributions of nicotine replacement therapy to areas where smoking is most common.

## Methods

The study protocol is published elsewhere.^4^ Briefly, general population survey data were used to select a region (specifically, a small town in Canada) with an estimated smoking rate of 35% over the two most recent survey periods.^1,5^ A letter offering five weeks of free-of-charge nicotine patches was mailed to every residence in the community (7257 households; mailed late October, 2020; 1 person per household could participate). The letter provided the choice of an online link or a telephone number to contact to make the request to be mailed nicotine patches. Each letter also had a unique ID number that needed to be entered as part of the nicotine patch ordering process (so each invitation could only be used once). At the same time, an article was published in the local newspaper to inform residents of the town about the ongoing project. In early December of 2020, a final newspaper article was published, offering the nicotine patches to any eligible residents of the town without the need to provide a unique ID number.

After consenting to take part in the study, participants were asked their age (needed to be 18 or older) and number of cigarettes smoked per day (10+ for a year or more). Participants provided their name, telephone number, and postal address. The 5-week supply of nicotine patches was couriered to them. Participants also agreed to take part in a 6-month follow-up asking about their smoking status at that point (payment of $20).

### Ethical Approval

This research project was approved by the standing ethics review committee of the Centre for Addiction and Mental Health (CAMH—REB Protocol #122/2019).

## Results

A total of 123 participants registered for the study and received nicotine patches (117 of these provided a unique ID, indicating that they responded to the invitation letter). Mean (SD) age was 48.6 (11.9), 59% were female, and 32% reported a gross family income of CAN$40 000 or less per year. Participants reported smoking an average (SD) of 19.5 (7.6) cigarettes per day at baseline. About three-quarters (72%, *n* = 88/123) completed the 6-month follow-up. Of those who completed the follow-up, 43.8% reported using all, and 18% reported using most, of the five-week supply of nicotine patches. Of the 123 participants, 18.7% (*n* = 23) reported 30-day point prevalence abstinence at 6-month follow-up (23.6% reported no smoking in the past 24 h and 8.9% reported no smoking since receiving the nicotine patches).

## Discussion

The majority of current smokers say that they would be interested in receiving free-of-charge nicotine patches to help them stop smoking.^6^ Further, previous research has demonstrated that prospectively contacted smokers participating in random digit dialing telephone surveys willingly have nicotine patches mailed to their household.^6^ However, the current study found that a postal mailed offer of free-of-charge nicotine patches targeted to a region with high smoking rates yielded only a limited number of participants actually requesting the patches. The potential benefits and feasibility of this targeted approach warrant additional expanded testing in additional jurisdictions.

The implementation of this project was limited by the COVID-19 pandemic, with plans to promote the distribution, including an onsite pick-up point, canceled due to the need for social distancing. This, in addition to possible concerns about the offer being a “scam”, might have reduced the overall response rate. However, it is probably reasonable to assume from these results that, even without the ongoing pandemic, it is likely that only a small minority of current smokers will proactively engage with an offer to be sent free-of-charge nicotine patches to help them stop smoking. Along with replication of this research once the pandemic subsides to determine if uptake can be increased, further research is merited on whether even this low level of engagement is cost-effective, and on whether more support (eg, additional patches or combined with other support services) would yield improved results.

## Supplementary Material

ntac244_suppl_Supplementary_Taxonomy-form

## Data Availability

Data is available from the corresponding author on reasonable request.
